# Phase lead/lag due to degree inhomogeneity in complex oscillator network with application to brain networks

**DOI:** 10.1186/1471-2202-16-S1-P127

**Published:** 2015-12-18

**Authors:** Junhyeok Kim, Joon-Young Moon, Uncheol Lee, George A Mashour, Seunghwan Kim, Tae-Wook Ko

**Affiliations:** 1 Nonlinear and Complex Systems Laboratory, Department of Physics, Pohang University of Science and Technology, Pohang 790-784, Republic of Korea; 2 Department of Anesthesiology, University of Michigan Medical School, Ann Arbor, MI 48109, USA; 3 Center for Consciousness Science, University of Michigan Medical School, Ann Arbor, MI 48109, USA; 4 National Institute for Mathematical Sciences, Daejeon 305-811, Republic of Korea

## 

**Figure 1:**
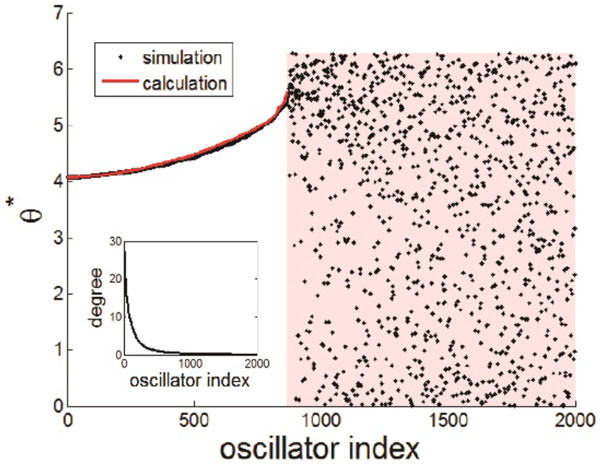
**The phase (black dots) of each oscillator and the calculated phases (red line) from analytic derivation**. They match well in locked region and red shaded area represents drifting region. The inset shows the degree for each node.

Brain anatomical connectivity is one of the main factors influencing information flow among the brain areas [1] and phase lead/lag relationship between oscillations of brain areas is known to be related to the information flow [2,3]. In this study, we analyze the network effect on the phases of coupled oscillators using Kuramoto model and obtain analytical relationship between phase lead/lag and degrees of network nodes. We also show robustness under various conditions, improving upon the result of ref. [4]. Using the brain anatomical connectivity and the relationship, we can explain the phase distribution across the brain. At first, we investigate the relationship in the oscillator model on a scale-free network of which degree distribution follows a power law [5]. Confirming the result of previous study [4], the phases of higher degree nodes lag the phases of lower degree nodes. Similar behaviors are observed also in random network, where the degree distribution follows a Poisson distribution. Using mean-field approximation, we analytically derive the relationship between phases and node degrees as shown in Figure 1. With various conditions of time delay and coupling strength, we also observe that this phase lead/lag relationship between nodes is robust. Our exact relationship can be well applied to human brain anatomical networks.
